# Carbolic Acid and the Treatment of Plague

**Published:** 1903-10-17

**Authors:** 


### CARBOLIC ACID AND THE TREATMENT OF PLAGUE.

It has been increasingly recognised of late that many of our most valuable drugs owe their good effects to the fact that they are antiseptics. Or, to be on the safe side, it may be said that the bactericidal properties of various potent medicines in general use have attracted more attention than they used to do, whether their beneficent properties in restoring health are due to their antiseptic action or not. Quinine, arsenic, mercury, and sodium salicylate are all drugs of the greatest value; they also possess a high degree of potency as antiseptics. The appreciation of this fact has led to the attempt to deal with infective diseases by raising the bactericidal power of the blood by the internal administration of antiseptics. It is obvious that whatever drug be used in this way it must be able to restrain the growth of the infecting organism without damaging to an equal extent the tissues of the patient. Until recently it was not thought possible that carbolic acid would fulfil these conditions, for it was not thought possible to give more than a few grains daily without causing danger. However, it has been shown that, to plague patients at any rate, very large doses indeed of carbolic acid may be given internally without giving rise to evil symptoms. Dr. Thomson,[1] in a report addressed to the principal medical officer, Hong Kong, upon the treatment of plague cases treated in the Kennedy Town Hospital up to July, 1903, gives a description of the various methods of treatment which have been in use from time to time in that institution, together with mortality tables arranged according to the treatment adopted. From these tables it appears that during the first half of the epidemic, before treatment by carbolic acid was used, the general case mortality was 85·6 per cent., while during the second half of the epidemic, when the patients were treated by large doses of carbolic acid, the case mortality fell to 36·4 per cent. The carbolic acid was given in doses of 12 grs. every two hours in a mixture flavoured with syrup of orange and chloroform water, 144 grs. of phenol being thus taken every 24 hours, and this treatment in some instances was continued over a long period of time. The treatment was continued so long as plague bacilli were present in the blood, a careful watch being kept against any symptoms of poisoning. One patient took in this way over 2,500 grs. of pure carbolic acid before his blood was free from plague bacilli.

In commenting upon the treatment Dr. Thomson states that symptoms of poisoning were practically unknown. In a few cases carboluria developed, but the omission of one or two doses was usually sufficient to clear the urine and permit the resumption of the remedy in full doses. Sometimes gastro-intestinal symptoms occurred, but greater dilution of the mixture with water was all that was required in these cases. In considering the figures given above showing the case mortality Dr. Thomson points out that there are two circumstances that need to be mentioned, and for which due allowance must be made—(1) The treatment with carbolic acid was commenced late in the epidemic, at a stage when there is a greater natural tendency to recovery, the disease being invariably more virulent early in the season; (*b*) Coincidently with his beginning the use of carbolic acid Dr. Bell announced his modification of Ross's method for the examination of a thick film of malaria blood as a method for the examination of plague blood, and as a consequence of this improved means of diagnosis a much larger number of very mild cases, many of which would not have been diagnosed as plague in former years, were proved to be plague and sent to Kennedy Town. These cases swelled the proportion of cases reported as recovering. However, as a result of observation of its use in a series of 143 cases Dr. Thomson considers carbolic acid in large doses the most hopeful means of treating plague thus far at disposal in Hong Kong, although it is by no means a specific remedy. While reporting thus favourably of its use he urges the desirability of carrying into effect before the next epidemic season certain proposals already sanctioned for the production locally of a curative serum for the treatment of plague, experience having proved that serum imported from Europe is useless.

It does not seem very likely that plague patients are specially resistant to the poisonous effects of carbolic acid, and it is more probable that the views which have been hitherto held regarding the poisonous properties of phenol are at fault. Perhaps, as has been suggested, when carbolic acid gives rise to symptoms of poisoning apart from any corrosive action, these symptoms are due to impurities and not to the phenol. However this may be evil symptoms and even death have been attributed, especially in the case of infants, to the absorption of comparatively small quantities of carbolic acid from wound surfaces, and even through the unbroken skin from preparatory dressings applied previous to an operation.

The problem is an important one, for if it be found that carbolic acid can be taken in such large doses as 144 grains daily in conditions other than plague it is possible that it may be a valuable remedy in other infective diseases.

[1] Journal of Tropical Medicine, Oct. 1.

